# Dichoptic vision in the absence of attention: neither fusion nor rivalry

**DOI:** 10.1038/s41598-019-49534-x

**Published:** 2019-09-09

**Authors:** Cheng Stella Qian, Sam Ling, Jan W. Brascamp

**Affiliations:** 1 Department of Psychology, Michigan State University, East Lansing, USA; 2 Department of Psychological and Brain Sciences, Boston University, Boston, USA; 3 Center for Systems Neuroscience, Boston University, Boston, USA; 4 Neuroscience Program, Michigan State University, East Lansing, USA

**Keywords:** Attention, Human behaviour

## Abstract

When the two eyes’ processing streams meet in visual cortex, two things can happen: sufficiently similar monocular inputs are combined into a fused representation, whereas markedly different inputs engage in rivalry. Interestingly, the emergence of rivalry appears to require attention. Withdrawing attention causes the alternating monocular dominance that characterizes rivalry to cease, apparently allowing both monocular signals to be processed simultaneously. What happens to these signals in this case, however, remains something of a mystery; are they fused into an integrated representation? In a set of experiments, we show this not to be the case: visual aftereffects are consistent with the simultaneous yet separate presence of two segregated monocular representations, rather than a joint representation. These results provide evidence that dichoptic vision without attention prompts a third and previously unknown mode, where both eyes’ inputs receive equal processing, but escape interocular fusion.

## Introduction

## Binocular Vision In The Absence Of Attention: Neither Fusion Nor Rivalry

Our minds are constantly presented with inconclusive and incomplete sensory information, from which it pieces together a unitary, conscious percept. One approach to understanding the mechanisms that support this ability is to interrogate the processing status of components that make up an ambiguous or conflicting stimulus, some of which reach awareness, and some of which do not. In this study, we leveraged a class of stimuli that evoke binocular rivalry^1^, wherein two disparate images are presented to the two eyes on corresponding retinal regions, resulting in perception of only one of the two images at a time, alternating over time. These fluctuations in subjective experience, despite invariant input, render binocular rivalry a valuable paradigm for studying visual awareness^2^.

It is generally agreed that awareness depends, to some degree, on attention: one tends to be aware of what one is paying attention to. The precise nature of this dependence, however, is a topic of active debate^3–5^. It is intriguing, therefore, that recent work suggests rivalry-induced fluctuations in awareness to necessarily require attention^6–8^. In particular, when an observer withdraws attention from stimuli that would otherwise cause rivalry, both monocular representations appear to receive equal processing. For instance, EEG signatures of both are simultaneously present^8^, and temporal regularities characteristic of rivalry alternations are absent^6^. While remarkable, this observation also presents a puzzle: if the two incompatible representations do not engage in rivalry, how, then, are they processed instead? In this study, we sought to better understand the fate of unattended, yet incompatible visual representations. In particular, we test the hypothesis that disparate monocular signals, in the absence of attention, are processed as if they were not competitive whatsoever, instead being fused into a combined cortical representation^8,9^.

How does one interrogate the processing status of unattended stimuli? The challenge is that observers cannot easily report on a display they ignore. To overcome this challenge, in this study we developed a novel paradigm that leverages well-established aftereffects in vision – aftereffects that reveal the nature of unattended representations. In our first experiment, we manipulated attention by including a demanding task at fixation, and assessed the fate of unattended binocular rivalry stimuli by examining their subsequent motion aftereffect (i.e. an illusory perception of motion following exposure to moving stimuli^10,11^). In our second experiment, we examined the fate of unattended, but fusible stimuli by examining their subsequent slant aftereffect (i.e. an illusory perception of slant following exposure to slanted stimuli^12^).

## Experiment 1a

One key novelty here is this: our binocular rivalry stimuli were designed to induce a motion aftereffect (MAE) in one direction if the monocular representations remain separate, yet in a different direction if they are fused into a joint ‘cyclopean’ representation. Specifically, each eye received a differently oriented, moving grating. In situations that involve no rivalry, these gratings both contribute to a combined MAE, and its direction is roughly opposite to the average direction of the two gratings (see Discussion for details). However, when these gratings are shown superimposed, without rivalry, then they form a *moving plaid* whose MAE direction is altogether different^13^. The central question, then, becomes: what MAE direction is observed after presenting these two gratings to separate eyes, and without attention? If such dichoptically presented images are fused under this condition, MAE directions should match that of the moving plaid, rather than that of the individual gratings.

### Methods

#### Participants

Eight participants were included in the data analysis (4 females and 4 males; age: *M* = 25.50, *SD* = 6.04, range 20–40). Two of the participants were authors (C.Q. and J.B.), while the remaining participants were students of Michigan State University who were naive to the purpose of the experiment. All naive participants were compensated at the rate of $10/hour and their informed consent were obtained before the participation. All experimental protocols were approved by the Institutional Review Board at Michigan State University and all experiments were performed in accordance with the approved guidelines and regulations. One participant performed the experiment but was excluded in the data analysis stage due to large within-subject variance across different sessions (see data analysis for details).

#### Materials

The experimental apparatus was a variant of the classical mirror stereoscope^14–16^ consisting of two mirrors (45° relative to participants’ midline) reflecting stimuli from two screens facing each other (62 cm away from the midline of the participant). A head rest stabilized the alignment of participants to view the reflection of one mirror with each eye.

Visual stimuli were displayed on two 24-inch flat-screen monitors (60-Hz refresh; mean luminance 31.8 cd/m^2^) as the only source of illumination in a dark testing room. All the stimuli were presented on a gray background. All aspects of the experiment were generated in MATLAB with the Psychophysics Toolbox^17–19^, running on a Mac Mini.

#### Stimuli

**Figure 1:**
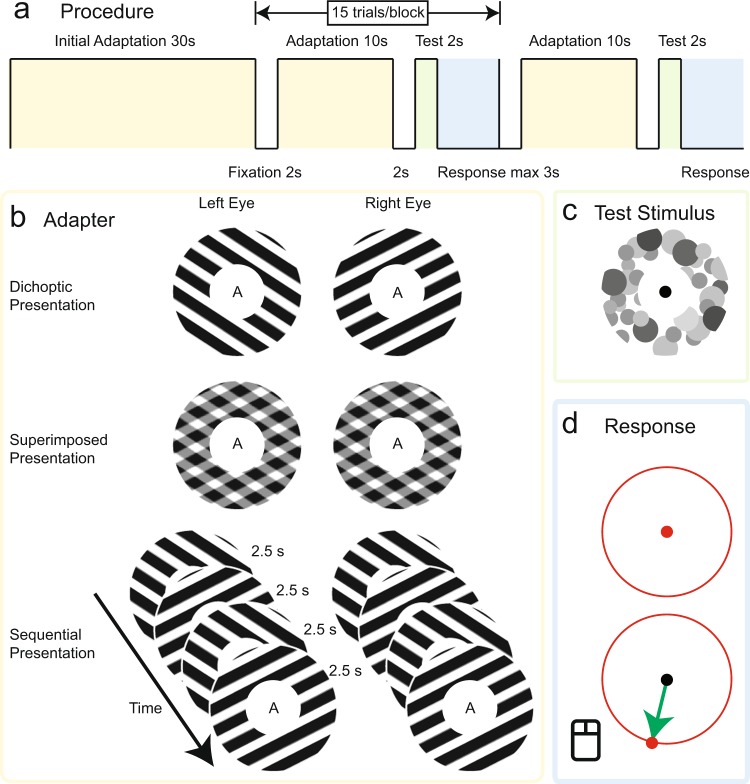
Stimuli and procedure of the experiment. (**a**) Procedure. Each block started with 30 seconds of adaptation and continued with 15 trials that included top-up adaptation and response. Each trial consisted of a fixation period, a short period of adaptation, a blank screen, a test stimulus, and the response period during which participants indicated the perceived motion direction of the test stimulus. (**b**) There were three adapter conditions: the Dichoptic Presentation condition, the Superimposed Presentation condition, and the Sequential Presentation condition. Participants performed each condition twice: once with attention to the adapter and once with attention to a central RSVP task. **(c**) The test stimulus consisted of dots which randomly varied in luminance, position, and size. (**d**) Response interface: a red dot appeared at the center and participants moved it with the computer mouse to report the direction in which the test stimulus was perceived to move. A nulling procedure, involving the addition of directional motion to the test stimulus, allowed us to estimate both MAE direction and MAE magnitude from these responses.

The gratings were tilted 15° clockwise and 15° counter-clockwise (see Fig. 1b Dichoptic Presentation for the appearance of the gratings). They were square wave gratings (spatial frequency: 1.50 cycles/°; Michelson contrast one; mean luminance same as the background) with slightly smoothed line edges to achieve anti-aliasing. The gratings were shaped as annuli with boundaries that faded into the background using a sinusoid function (gradients between 6.40–9.54° and between 1.61–2.40° in eccentricity; background luminance: 35 cd/m^2^). One of the gratings moved down, orthogonal to its orientation, at a speed of 1.35°/second, and the other grating also moved down, orthogonal to its own orientation, but four times as fast. The speed assignment between the two gratings was counterbalanced across blocks, as was the eye assignment.

Two other conditions involved the same gratings but not presented dichoptically. Instead, these conditions provided baselines to which to compare the results of our rivalry condition. The ‘superimposed’ presentation condition had the two gratings averaged in luminance to become a plaid (Fig. 1b), shown identically to both eyes. The aftereffect in this condition indicates which aftereffect direction can be expected if these gratings form a fused representation. The second, ‘sequential’ presentation condition had the two gratings presented the same to both eyes, but in alternation for 2.5 seconds at a time. The aftereffect in this condition shows which aftereffect direction can be expected if these gratings are processed independently. It is important to note that even though this latter condition involved an ongoing alternation between the two component gratings on the screen, the MAE direction in this condition is not specifically a signature of rivalry-like alternations; instead, it is a signature of independent processing of the two gratings which, for practical reasons, was achieved through their temporal separation in this control condition.

Given the stimulus orientations and speeds we selected for these stimuli^13,20^, we expected the MAE direction for the sequential condition to be near opposite to the average direction of the two individual gratings, i.e. straight up (the speed difference between gratings does not strongly affect this). For the superimposed condition, we expected a MAE direction that deviates from straight up in the direction opposite to the direction of the faster grating. Previous work has demonstrated that this type of surprising disconnect between the MAE direction of a plaid and that of its component gratings does not require the two component gratings to be presented to the same eye; an observation on which our current approach is built^21–23^.

The test stimulus and response interface were built around a novel method that we developed to measure the direction and magnitude of a MAE simultaneously. The test stimulus (see Fig. 1c) was a dot field consisting of dots of various sizes (0.5 to 1°) and luminance (26.2 to 57.6 cd/m^2^). This design was intended to achieve stimulus energy at a broad range of spatial frequencies and orientations (thus allowing motion aftereffects in all directions) while maintaining an average luminance similar to the adapter and the background. The test stimulus could be stationary or coherently move in a certain direction (see procedure for details). The response interface, presented after the termination of the test stimulus, consisted of one black fixation point, one response boundary that outlined where the test stimulus had been, and a red dot controlled by the computer mouse (see Fig. 1d). During the response phase, participants could move the red dot to indicate the MAE direction they perceived during the test stimulus phase. The direction was recorded once the center of the red dot overlapped with the response boundary. The response interface terminated upon a participant’s response or after 3 seconds has passed. In the latter case one more trial was added to ensure 15 trials with response for each block. All participants completed each block within 18 trials.

In addition to the three adapters (dichoptic, superimposed and sequential), there were two attention conditions. In the ‘attended’ condition, participants were instructed to report a small contrast decrement lasting 100 ms in the adaptation stimulus itself with a key press response. The contrast decrement appeared randomly in time but on average once every 10 seconds during both initial adaptation and top-up adaptation (see more details in Procedure). The magnitude of this contrast change was controlled online via a staircase aimed to converge at a hit rate of 75% (using BEST PEST as the fitting procedure conducted with Palamedes Toolbox^24^), thus ensuring a demanding task. In the second attention condition, termed the ‘unattended’ condition, the task was to report the letter X in a rapid serial visual presentation (RSVP) stream at fixation, directing attention away from the adaptation stimulus, by pressing another key. The difficulty of this task was also controlled online by titrating the RSVP presentation rate, using a staircase procedure aimed to converge at a 75% hit rate. Both the contrast changes and RSVP stream were on the screen during both attention conditions, but only one of them was task relevant at a time.

#### Procedure

A generic block started with an adaptation period of 30 seconds, during which participants responded to the RSVP stream or contrast change according to the task assigned (see Fig. 1a). There were 15 trials after the initial adaptation. Each trial started with a top-up adaptation (10 s), with the same stimulus and task as during the initial adaptation period. The targets for both the RSVP task and the contrast detection task were not presented in the first 500 ms and the last 1000 ms of each adaptation period to make sure participants had enough time to be prepared and respond to the target. During initial adaptation one target was presented during each consecutive 10-second period (3 targets total), and a single target was presented during each period of top-up adaptation. After top-up adaptation, the trial continued with a blank screen with a fixation point for 2 seconds. This blank was specifically intended to mitigate short-term effects of the most recent 2.5-s grating in the sequential condition, to make sure that the test stimulus more accurately measured the overall adaptation effect produced by both gratings. The test stimulus was then shown for 2 seconds and the trial ended with the response interface.

**Figure 2:**
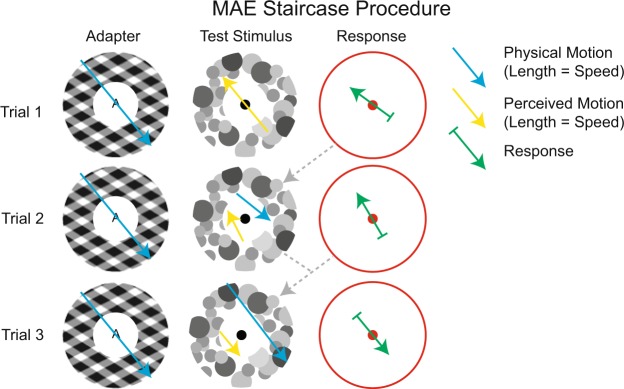
MAE nulling procedure. The goal of the nulling process was to capture the magnitude and the direction of the MAE at the same time. The first trial of the experiment started with a physically static test stimulus. After the response of the participant, the test stimulus on the second trial moved in the direction opposite to the first trial’s response, to compensate for the MAE. The motion of the test stimulus on each following trial was determined as the vector sum of the test stimulus’ motion on the previous trial and the participant’s response direction on that last trial. The length of this latter vector was systematically reduced from trial to trial to achieve higher precision (smaller adjustments) in later trials.

We devised a novel nulling procedure to measure the direction and the magnitude of the MAE at the same time. As with existing MAE nulling methods, the overall goal was to adjust the movement of the test stimulus to compensate for the MAE, so that the test stimulus appears static^25,26^. The difference with other nulling methods, however, is that this procedure provides estimates of two variables, speed and direction, at the same time. During the first trial the test stimulus was actually static, but for every trial after that, its speed and direction were calculated as the vector sum of its speed and direction on the previous trial (one vector) and the direction opposite to the observer’s MAE response on that previous trial (the other vector; see Fig. 2). The length of the first vector represented speed, but the length of the second vector (the one encoding the observer’s response) was arbitrarily set to start as equivalent to 0.15 degrees/s on the first trial, and then to reduce by a factor 0.8 on each subsequent trial to achieve more precise adjustment in later trials. In other words, the trial-to-trial adjustment of the test stimulus was coarse and dramatic in earlier trials but precise and subtle in later trials. This same trial-to-trial reduction in the weight of the response vector also ensured that, even though observers never reported perceived speed (only perceived direction), this procedure converged, across repeated trials, on a motion vector that represents both direction and magnitude. By way of illustration, consider the second trial of a given condition: the trial during which the test stimulus contains nulling motion in the direction exactly opposite to the motion direction reported during the first trial, and at a speed of exactly 0.15 degrees/s. Consider a case where reported MAE direction on this second trial is the same as the direction that was reported on the first trial, during which the test stimulus was physically stationary. This indicates that 0.15 degrees/s is insufficient to cancel out the MAE, and on the third trial our procedure ensures a nuller that has the same direction as it did on the first trial, but that moves faster (1.8 times as fast). In contrast, consider another case where reported MAE direction on the second trial is exactly opposite to the direction reported on the first trial. This indicates that a nuller that moves at 0.15 degrees/s overpowers the MAE, and our procedure ensures a weaker nulling motion (0.2 times as strong), but again in the same direction, on the third trial. This same principle, acting across many trials, leads to a nulling motion vector that cancels the MAE in both direction and magnitude.

Observers were instructed to randomly guess a motion direction if they perceived no net motion in the test stimulus. This might result in inaccurate nuller stimuli during the course of a staircase procedure but, again due to the ever-reducing weight of every consecutive response vector, is not expected to lead to systematic deviations in the staircase endpoint.

Before the main experiment, each participant completed 2 sessions of the superimposed presentation condition as a pre-screening. Each pre-screen session had 4 blocks of the attended condition and 4 blocks of the unattended condition. The purpose of these pre-screen sessions was to ensure that each observer’s MAE in the superimposed condition was distinct from straight upwards; that is, distinct from what we expected in the sequential condition. This was critical because those two conditions formed the two reference points to which our condition of interest would be compared to distinguish whether its stimuli behaved like an integrated plaid or as separate components. To screen for this precondition, the two pre-screen sessions differed in terms of the motion direction of the stimulus, with the faster grating moving either to the lower right (presumably producing a MAE to the top left) or the lower left (presumably producing a MAE to the top right). If the resulting motion direction was, indeed, significantly different between these two sessions then the observer passed the screening. Three observers were excluded on these grounds. An observer was excluded after the main experiment because their MAE direction in the superimposed condition significantly differed between the pre-screening sessions and the main experiment.

A practice session was conducted after the pre-screening but before the main experiment. The stimuli of the dichoptic presentation condition were shown on the screen and every participant reported seeing at least 2 switches in 3 consecutive top-up adaptation phases. Transitions between two precepts in a mosaic fashion were reported but no participant reported seeing a plaid percept. Indeed, plaid precepts typically only occurs very briefly with low contrast stimuli^27^ or flickering stimuli^28^. The observers were also encouraged to verbally report if they saw any plaid percept during the attended dichoptic presentation condition but no participant reported any.

To determine statistical significance for the MAE direction data based on the staircase results, we could not use traditional statistical methods, e.g. a repeated measures ANOVA. Those methods require the calculation of an arithmetic mean, but the actual mean angle we acquired is the geometric mean, i.e. based on the vector average across all observers’ MAE vectors. This ensures that a vector that reflects an individual’s MAE contributes less to the average angle computation if its length, the MAE speed, is smaller, in keeping with the fact that, using our nulling procedure, the angle estimate is more subject to noise in the case of a smaller vector than in the case of a larger vector (see Methods for further details). To determine the statistical significance of the difference in MAE direction between conditions, we conducted a Monte Carlo simulation based on the Cartesian coordinates of the vector endpoints. Specifically, we calculated the population distribution of endpoint x values and of endpoint y values by calculating traditional means and standard deviations for each condition, and then numerically computed a distribution of angles by randomly drawing from the resulting endpoint distributions. In other words, each simulated angle in this Monte Carlo procedure was obtained by randomly drawing a pair formed by one x value and one y value from the two distributions and computing the corresponding angle. After estimating the standard error on our angle measure in this fashion, the angles of each condition could be compared with traditional t-tests. In addition to this analysis on the outcome of the staircase procedure, we also performed a traditional repeated measures ANOVA on the initial MAE directions reported on the first trials of the experiment blocks (when the test stimulus was physically stationary). The issues described above do not apply to this measure because there is no vector length in play.

### Results

We found that some participants had a systematic response bias in the sense that the reported MAE direction, when averaged across two mirror reversed versions of a given adapter (i.e. the versions with the faster grating moving either to the bottom left or to the bottom right), was not vertical. To compensate for this response bias, we interpreted each observer’s average MAE direction across all conditions combined as their ‘subjective vertical’. All MAE directions of a given observer were then rotated by the angle that would align their subjective vertical with objective vertical. In addition, in our subsequent analyses MAE angle was mirrored in the (newly calculated) vertical for one of the two adapter versions, so that we could collapse across both versions (i.e. the ones with the faster grating moving either to the bottom left or to the bottom right).

**Figure 3:**
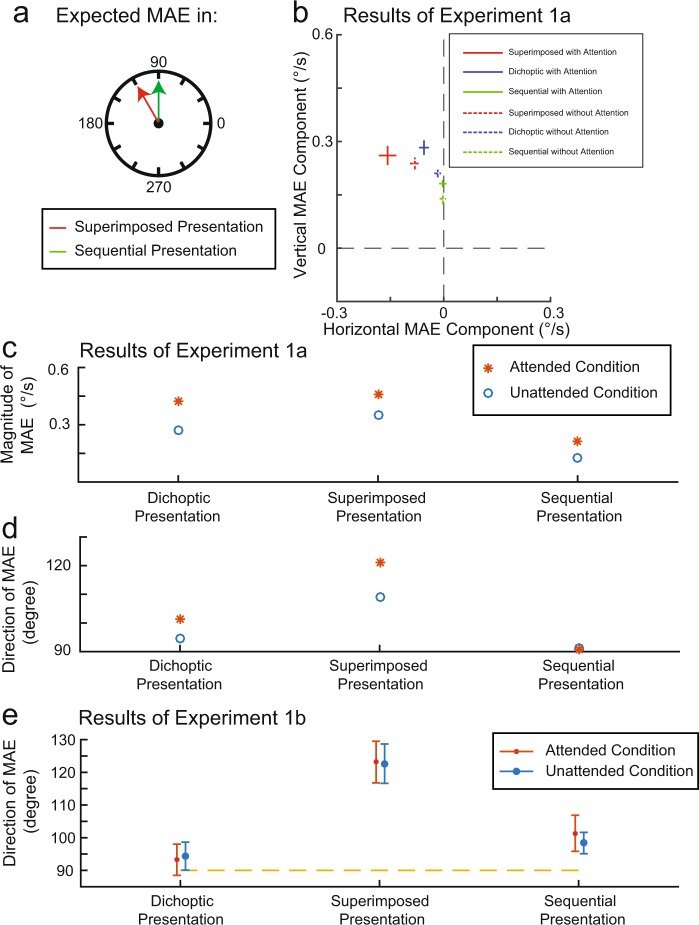
Results of Experiments 1a and 1b. (**a**) The expected MAE directions of the superimposed presentation condition and the sequential presentation condition. Panel (b) shows the MAE results for different conditions in Cartesian coordinates across 8 participants. The vector from the origin to the center of each cross denotes the MAE for each condition, with length representing aftereffect magnitude and angle representing aftereffect direction. The bars forming each cross are within-subject standard error following the method of Loftus and Masson^56^. The magnitude (**c**) and angle (**d**) of the motion aftereffect (MAE) for each condition. Panel c and d are results acquired from the geometric mean. Error bars based on the arithmetic mean are provided in panel b. (**e**) MAE direction in Experiment 1b. The error bars denote within-subject standard error following the method of Loftus and Masson^56^.

In Fig. 3b the MAEs in different conditions (as inferred from the staircase convergence points) are denoted as crosses that mark the horizontal (x-axis) and vertical (y-axis) components of the motion aftereffects (see Procedure and Fig. 2 for the methods of acquiring both magnitude and angle of the MAE). The bars that form each cross indicate the within-subject errors, i.e. the variability due to subject by condition interaction. To ensure our attention manipulation was successful we first analyzed MAE magnitude (see Fig. 3c), which in all adapter conditions should be reduced when attention is withdrawn^29–32^. We conducted a repeated-measures ANOVA with two factors (Adapter, 3 levels; Attention, 2 levels). The magnitude was significantly higher in the attended than the unattended condition, indicating that the attention manipulation was successful (see Supplementary Table for detailed statistics for this test, as well as for several of the test reported in the remaining text). A post hoc comparison also showed that the sequential presentation condition produced weaker MAEs than the other two conditions, possibly due to the constant changes of the adapter stimulus^33^. The interaction between attention and adapter was not significant.

Our main focus was the direction of the MAE, as an index of the processing status of the adapters. As a first step, we performed a repeated-measures ANOVA on the horizontal component of the MAE (i.e. the x-coordinates of the plus signs in Fig. 3b), to verify that, as intended, the sequential and superimposed reference conditions differ in the extent to which the MAE deviates from vertical (factors: Adapter, 3 levels, and Attention, 2 levels). Indeed, the horizontal component showed a significant adapter effect and a post hoc analysis confirmed that the horizontal component of the MAE was smallest in the superimposed presentation condition and largest in the sequential presentation condition. The dichoptic presentation condition fell in between these two conditions and significantly differed from both (when including both attention conditions). The vertical component of the MAE (i.e. the y-coordinates of the plus signs in Fig. 3b) primarily demonstrated the magnitude effect described in the previous paragraph and showed statistical patterns similar to those described there (see Supplementary Table for detailed statistics).

An ANOVA performed on the horizontal MAE component (see above) showed no effect of attention nor an interaction between attention and adapter condition, as might have been expected if the two dichoptic gratings had formed a fused plaid in the absence of attention. In fact, a post hoc test showed that the difference between the dichoptic condition and sequential condition, although significant in the attended condition, actually disappeared in the unattended condition, while the difference between the dichoptic condition and the superimposed condition remained significant and became numerically larger. This provides a first piece of evidence against the idea that a dichoptic plaid starts to behave more like a superimposed plaid when attention is withdrawn.

To investigate the influence of attention on MAE angle directly, rather than using the horizontal MAE component as a proxy, we used a Monte Carlo simulation (see methods; an ANOVA was not possible for technical reasons: The target variable (e.g. direction of MAE in this paper) in an ANOVA is assumed to conform to a normal distribution, so that the mean and standard deviation of the sample can be used to infer other statistical properties. However, while the mean of the dataset in an ANOVA refers to the arithmetic mean, the actual mean angle we acquired is the geometric mean, based on the vector average across all observers’ MAE vectors. This ensures that a vector that reflects an individual’s MAE contributes less to the average angle computation if its length, the MAE speed, is smaller. Instead of performing an ANOVA, we conducted a Monte Carlo simulation based on the horizontal and vertical components of the MAE, because the horizontal and vertical components of the MAE do conform to a normal distribution.) to calculate the statistical differences in MAE angle among unattended conditions only. Critically, the unattended dichoptic condition did not show a significant difference with the unattended sequential condition (*p* = 0.42), but did show a significant difference with the unattended superimposed condition (*p* < 0.01). This again indicates that the dichoptic stimulus, when unattended, behaves like two independent gratings and not like an integrated plaid. Applying the same Monte Carlo approach to the attended conditions showed significant angle differences between the dichoptic and the sequential conditions (*p* = 0.02), as well as between the dichoptic and superimposed conditions (*p* = 0.002; see Discussion). Taken as a whole, these results indicate that the unattended dichoptic presentation condition did not show any signs of being different from the sequential presentation condition, yet did differ significantly from the superimposed condition.

As a final examination of our main question, we examined the angle of the first MAE reported in each experiment block, when the test stimulus was still physically standing still. As outlined above, this initial MAE angle does lend itself to a repeated-measures ANOVA (factors: Adapter, 3 levels; Attention, 2 levels), rather than depending on pairwise tests. The attention effect nor the interaction was significant. The adapter effect was significant, showing that the dichoptic presentation condition gave rise to a MAE angle that is less vertical than the angle of the superimposed presentation condition but higher than the angle of the sequential presentation condition (again see Supplementary Table for detailed statistics). More importantly, the critical comparison is between the unattended dichoptic condition and the other two unattended conditions. A one way repeated-measure ANOVA between the 3 unattended conditions showed a significant main effect, and a post hoc analysis showed that the unattended superimposed condition had a significantly higher MAE angle than both the unattended dichoptic and the unattended sequential presentation conditions individually, whereas the latter two were not significantly different.

### Conclusion and discussion of experiment 1a

Converging evidence across three analysis approaches shows that MAE direction in the unattended dichoptic presentation condition, our critical condition, was significantly different from that in the superimposed presentation condition, but similar to that in the sequential presentation condition. This provides evidence that, in the absence of attention, conflicting information from the two eyes is not fused, at least not at the level where our aftereffect arises.

## Experiment 1b

One possible interpretation of this result is that dichoptic input without attention is fused after all, but that this happens at a level that is later than the level at which our MAE arises^34^. With that in mind we designed a control experiment that made use of a different MAE, one that is thought to arise at a later cortical processing stage than the MAE of Experiment 1a. In particular, given the motion content of the adapters (relatively slow) and test stimuli (almost stationary) of Experiment 1a, its MAE would be considered a ‘static MAE’, thought to arise relatively early, possibly in V1^11^. One criterion in deciding the processing level associated with a MAE is the degree of interocular transfer, meaning the degree to which an adapter presented to one eye affects perception of a test stimulus in the non-adapted eye. If a MAE has complete interocular transfer, this provides evidence that it arises at binocular processing stages. Importantly, static MAEs show only partial interocular transfer^11^, implying that they may partly arise prior to interocular combination. For Experiment 1b, therefore, we aimed for a MAE that is known to have full interocular transfer and that, therefore, plausibly arises after interocular combination. Otherwise we kept the approach the same as Experiment 1a. In particular, we increased the speed of motion in the adapter and changed the test stimuli to be a flickering noise pattern in order to capture the so-called ‘dynamic MAE’^35^. The dynamic MAE has full interocular transfer^36^, consistent with evidence that it arises in MT^37^, where neurons tend to respond to moving stimuli presented to either eye^38–41^.

### Participants

Four participants participated in this control experiment. They were all included in the data analysis (2 females and 2 males; age: *M* = 28.25, *SD* = 6.50, range 25–40). All of them participated in Experiment 1a.

### Stimuli and procedure

The adaptation stimuli were kept the same as in Experiment 1a, except the speed of the moving gratings. The speed was determined by a pre-test for each participant. The pre-test had each participant perform the superimposed presentation condition with three different speeds, and with our altered test stimulus, intended to elicit a dynamic MAE (see below). The speed that was chosen was the one that yielded the least variance in reported MAE direction across trials. Two participants ended up with 2.03°/second for the slower grating and the other two with 3.38°/second. The other grating moved 4 times as fast as the slower one.

The test stimulus here was changed to a flickering pixel noise pattern (pixel size: 0.03°, mean luminance: 31.8 cd/m^2^, standard deviation: 15.9 cd/m^2^, flickering frequency: 30 Hz) to better capture the dynamic MAE^11^. Having already established in Experiment 1a that these participants could comply with the attention instruction, we were less interested in MAE magnitude this time, so the test stimulus was kept the same for every trial and never contained any net directional motion. Corresponding with this, instead of estimating a MAE nulling vector, we collected 8 direction responses for each condition and performed a different statistical analysis than for Experiment 1a.

On 6.25% of the trials (12 trials total), observers reported a MAE that moved downwards. Given that all the motion in the adapters was downward (both the component gratings and the plaid), these responses were taken as mistakes and excluded from further analysis.

### Results

The MAE direction for each condition is shown in Fig. 3e. A single-trial analysis was conducted with linear mixed-effects models^42^. LME models allow us to account for all the responses participants made rather than collapsing per observer-condition combination (i.e. within-subject variability is included in the model as a random effect). We included attention (two levels) and adapter (three levels) as fixed factors. Neither the attention effect nor the interaction between attention and adapter was significant, but the adapter effect was (see Supplementary Table for detailed statistics). A post hoc analysis showed that in the superimposed presentation condition the MAE had a significantly less-vertical angle than in the other two conditions, consistent with the results of Experiment 1a. To more closely examine the fate of the dichoptic stimulus in the unattended condition, the same analysis was conducted for the three unattended conditions alone with the adapter as the fixed factor. The adapter effect was significant and the post hoc analysis showed that the unattended superimposed condition had a significantly less vertical MAE angle than each of the other two unattended presentation conditions, but that there was no significant difference in MAE angle between the unattended dichoptic condition and the unattended sequential condition.

### Conclusion and discussion of experiment 1b

We found that MAE direction in the unattended dichoptic presentation condition, which is our critical condition, was significantly different from that in the superimposed presentation condition, but indistinguishable from that in the sequential presentation condition. This further bolsters the interpretation that in the absence of attention, conflicting information from the two eyes is not fused.

## Experiment 2

The first two experiments suggest that an exceptional situation arises when dichoptic stimuli are viewed but not attended: the monocular signals do not engage in rivalry, but they are not fused either. To investigate whether this remarkable situation is unique to unattended dichoptic situation or, alternatively, whether it applies to unattended binocular stimuli generally, we performed a final experiment examining the fate of *non-*conflicting binocular input in the absence of attention. Two compatible monocular images can clearly be fused when attention is applied, but it remains unclear whether they still fused in the absence of attention. Can normal interocular fusion be abolished by inattention?

To test whether interocular fusion relies on attention, we exploited slant aftereffects (SAE). SAE occur after adaptation to a slanted surface: a surface that is physically upright and perpendicular to the line of sight, appears slanted in the direction opposite to that of the adapting surface^12^. We replaced the adapting stimuli from our first two experiments with a surface that had slant from interocular disparity (i.e. each eye viewed the same grating from a slightly different simulated viewpoint) and measured whether its resulting SAE could still be observed when attention was withdrawn from this adapter. If so, this would indicate that fusible information from the two eyes is combined as normal in the absence of attention, even if non-fusible information is not.

### Participants

Eight participants finished the experiment and were all included in the data analysis (4 females and 4 males; age: *M* = 27.13, *SD* = 6.63, range 20–40). Four of them participated in Experiment 1a and three participated in Experiment 1b. Two other participants reported not seeing any slant in the adaptation stimulus itself and, as a result, were not asked to finish the experiment.

### Stimuli and procedure

**Figure 4:**
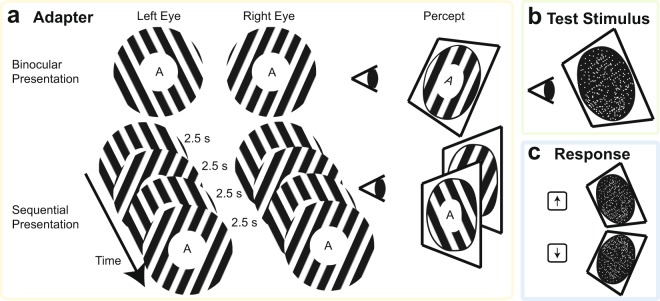
The design of Experiment 2. The procedure of Experiment 2 was the same as that of Experiment 1a. (**a**) The adapters were presented in the same fashion as the MAE adapters in Experiments 1 and 2. The binocular presentation condition has two gratings presented to two eyes, and the interocular disparity gives rise to perception of an approximately vertical grating that is rotated in depth around its central horizontal axis (i.e. slanted). The sequential presentation condition involves the same two images as the binocular presentation condition but presented sequentially. Observers did not experience a slant in this condition. (**b**) The test stimulus was a random dot stereogram that varied from trial to trial in its degree of slant from disparity. The angle of this slant was adjusted using a staircase designed to converge on subjective upright. (**c**) Participants were instructed to respond to the perceived direction of slant in the test stimulus.

The SAE adaptation stimulus was based on the original MAE adaptation stimulus of Experiment 1. Specifically, a vertical square wave grating that had the same spatial frequency as that original grating was now shown from a different simulated viewpoint in each eye, corresponding to a striped surface that was slanted by 60° relative to vertical (in either direction). As a result of the viewpoint difference, the individual monocular images were actually slightly tilted in opposite directions relative to vertical (see Fig. 4a Binocular Presentation), and the actual on-screen spatial frequency was slightly different between the top and bottom of each image.

Like Experiment 1, this experiment also included a control condition involving sequential presentation of the two gratings that made up our slanted adapter (see Fig. 4a Sequential Presentation). In this control condition no slant from binocular disparity was present, and it served the purpose of controlling for any monocular cues to depth that might be present in the individual gratings and that might cause a SAE without interocular combination. Critically, however, we were interested in whether the unattended binocular presentation condition could induce an SAE. To minimize negative afterimages the adaptation stimuli in both conditions shifted by one fifth of a period to either left or right every 2.5 seconds.

Each test stimulus was a random dot stereogram (each dot 0.2°; around 300 dots). The degree of slant from disparity present in this stereogram was adjusted from trial to trial with a staircase set to converge on the subjective upright: the observers’ perceptual experience of a surface vertical to the line of sight. Achieving slant from disparity involved slight deformations in the monocular random dot displays that also resulted in a dot density gradient along the vertical axis. We counteracted this gradient to, again, achieve a homogeneous on-screen density for each slant, in order to keep monocular depth information in the test stimulus to a minimum. Note that this random dot test stimulus filled the whole circular aperture, whereas the adapting gratings filled an annulus region in which the center part was kept open to allow space for the RSVP stream. This did not seem to affect the perception of the test stimulus, since no observer has reported any difference in perceived slant between the central and the surrounding region in the test stimulus.

The temporal structure was the same as in Experiment 1a (see Fig. 1a). After the presentation of the test stimulus the fixation turned red, and participants pressed one of two keys to indicate whether the top or the bottom looked closer to them (see Fig. 4c). The degree of slant in the test stimulus at the end of the procedure formed our measure of the SAE.

The procedure of this experiment was also modeled after that of Experiment 1a in other regards (see Fig. 1a): each participant finished 8 blocks during each session with a factorial design of two presentation conditions (binocular and sequential) by task (attend contrast changes in the adapter and attend RSVP task). The two sessions differed in the sign of the adapter’s slant. Unless otherwise mentioned, the procedure of the Experiment 2 was kept the same as that of the Experiment 1 to maximize comparability.

As in Experiments 1a and 1b, systematic bias was removed from individual-observer data before performing group-level analyses. In particular, and analogous to the approach described above for MAEs, the average of all the SAE results of a given participant (i.e. pooling across trials in which the adapter slants had opposite signs) was taken as subjective upright, and all reported slants were rotated by the angle required to align subjective upright with objective upright.

### Results

**Figure 5:**
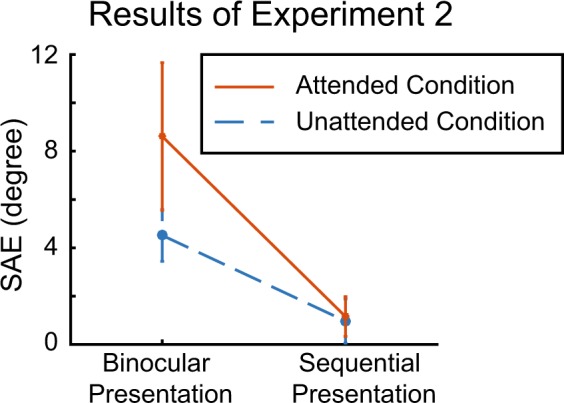
The results of Experiment 2. Both Binocular presentation conditions have a significant SAE, while the sequential presentation conditions do not.

The SAE results across participants are shown in Fig. 5. We conducted a repeated-measures ANOVA with two factors (Adapter, 2 levels; Attention, 2 levels) and found a significant adapter effect but neither an attention effect nor an interaction (the numerical trend toward an interaction that is visible in the figure is not significant: *p* = 0.16). A post hoc test showed that the binocular condition has a higher SAE than the sequential presentation condition (see Supplementary Table for detailed statistics). Independent sample t-tests were conducted to compare each condition with 0° of SAE. As expected, neither the attended nor the unattended sequential presentation condition produced a significant SAE (attended: *t* (7) = 1.51, *p* = 0.18, Cohen’s *d* = 0.53; unattended: *t* (7) = 1.13, *p* = 0.30, Cohen’s *d* = 0.40), confirming that the SAE in this experiment requires interocular interactions. More importantly, both the attended and the unattended binocular presentation condition gave rise to a significant SAE (attended: *t* (7) = 3.01, *p* = 0.02, Cohen’s *d* = 1.06; unattended: *t* (7) = 4.51, *p* = 0.003, Cohen’s *d* = 1.60), and the strength of the SAE was not significantly different between the two binocular conditions (*t* (7) = 1.54, *p* = 0.17, Cohen’s *d* = 0.54). Note that this latter result, a lack of effect of attention on the SAE, is not the main focus of this experiment. Instead, our main result is that we found a significant SAE in the unattended binocular condition (*t* (7) = 4.51, *p* = 0.003, Cohen’s *d* = 1.60) and a significantly higher SAE in the unattended binocular condition than in the unattended sequential presentation condition (*t* (7) = 2.68, *p* = 0.03, Cohen’s *d* = 1.35), which means that the unattended binocular presentation condition still gave rise to a SAE.

### Conclusion and discussion of experiment 2

The unattended binocular presentation condition still gave rise to a SAE, indicating that two compatible monocular images can still generate depth information in the absence of attention. Putting this in the context of our first two experiments, this experiment shows that only non-compatible monocular images fail to be integrated in the absence of attention (nor do they engage in rivalry), while compatible images still show signs of an integrated representation, regardless of whether the observer attends to them.

## General Discussion

Although both attention and binocular rivalry have been studied extensively, it was discovered only recently that binocular rivalry requires attention^8^. While this finding, corroborated several times since then^6,7,43^, suggests a surprisingly close association between rivalry and attention, it also raises new questions. Here we addressed perhaps the most pressing one: if unattended dichoptic inputs do not elicit rivalry, how are they processed instead? We tested a plausible, and previously suggested, candidate answer to this question^8,9^: that the two monocular streams are combined into a fused representation. Our results do not align with this hypothesis, instead indicating that the two monocular streams are not fused into a joint cortical representation (Experiment 1), and that this failure to fuse under inattention is unique to incompatible, as opposed to compatible, monocular inputs (Experiment 2).

What, then, is the processing status of unattended binocular rivalry stimuli? Although the existing evidence suggests that both images receive a similar amount of processing simultaneously^6,8^, here we show that this is not because they are fused into a joint representation. Besides fusion, we see two other possibilities: independent processing and patchwork rivalry. Regarding the first of these options, perhaps the two representations exist side-by-side in the processing hierarchy. The main difficulty with this idea is that eye-of-origin information is most prominent in early visual cortex^41,44,45^, and it is difficult to see how both representations can coexist at stages where neurons respond to input from either eye. One potential solution is that a separation in represented depth may allow the two monocular streams of information to be preserved side-by-side^22^, but we see no strong evidence for this.

This leaves the second possibility: patchwork rivalry. This corresponds to a situation in which rivalry is resolved locally, but in which eye dominance differs across different parts of the stimulus. Patchwork rivalry happens to some extent during attended binocular rivalry^46,47^ and our tentative hypothesis is that lack of attention increases this extent by affecting binding across retinotopic space. Zhang *et al*.^8^, the first study to report that rivalry requires attention, also considered this scenario, but opted for the fusion hypothesis based on indirect evidence. We do note that their experiment was slightly different from ours in terms of visual stimuli, and also in terms of measurement methods (electro-encephalography vs. aftereffects), so it is possible that their conclusion is not in conflict with ours. On the other hand, the basic finding that binocular rivalry is abolished by inattention has proven quite robust to changes in stimuli and methods, and has been corroborated using electrophysiological^7,8^, behavioral^6,43^ and neuroimaging method methods^48^, as well as using a wide range of stimulus parameters^6–8^.

By showing a difference between the ways binocular rivalry and stereopsis depend on attention, the present results indirectly bear on an existing segment of the literature that examines the relation between these two modes of binocular interaction. One possibility that has been forwarded in that context is that binocular rivalry and stereopsis are two separable processes that are completed independently before their interaction^49,50^. The findings of the current study are consistent with this idea, in that they show the absence of attention to differentially affect rivalry between dichoptically presented images and stereopsis based on fusible images. Moreover, the findings suggest that inattention can weaken the representation of both dichoptic and fusible input (as evidenced by MAE strength and SAE strength, respectively), but does not allow dichoptic input to enter a fused state.

**Figure 6:**
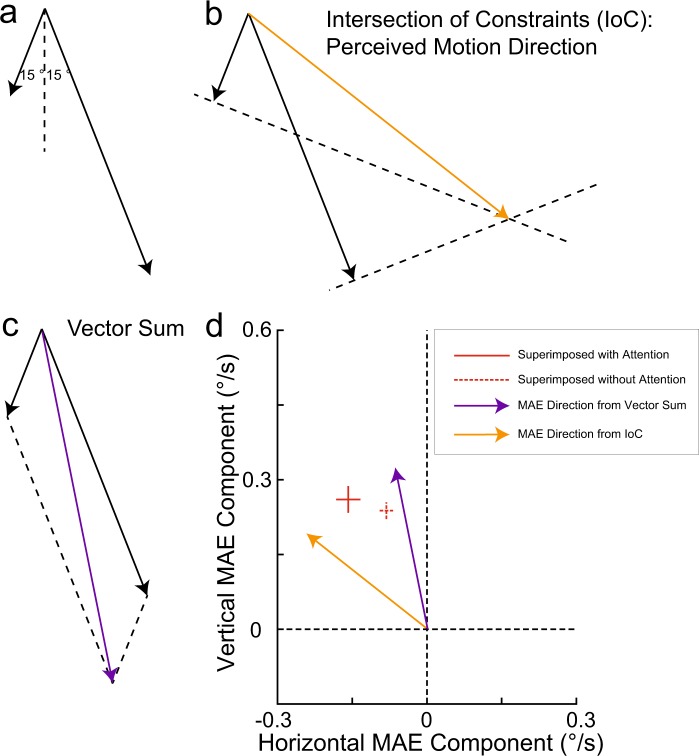
Schematic demonstration of the direction of vector sum and intersection of constraints. (**a**) The direction and speed of the two adapting moving gratings. The length of the vector denotes the speed of the motion. (**b**) Demonstration of the direction from intersection of constraints. The two dashed lines are perpendicular to the two motion vectors. Each line denotes the endpoints of all possible motion vectors that are consistent with the observed motion of one of the gratings. (**c**) Demonstration of vector sum. (**d**) The MAE directions from vector sum and intersection of constraints presented in the same figure with the MAE we measured in the attended and unattended superimposed presentation conditions.

Our Experiment 1a showed two data patterns that are not central to our question but that warrant discussion. First, in the dichoptic adaptation condition, the MAE angle deviated away from vertical more in the attended condition than in the unattended condition, which might be because inattention can abolish binocular rivalry. Rivalry does happen in the attended condition, and it might be expected that our fast-moving grating was perceptually dominant for longer than the slow-moving grating^51^. This, in turn, would result in the fast-moving grating contributing more to the combined MAE^52^, consistent with the observed deviation from vertical in the attended condition. In the non-attention condition, because of a lack of rivalry, such an argument would not apply. The second data pattern is that the MAE angle for the superimposed stimulus also showed a small but significant difference between the attended and the unattended condition (see Figs 3b and 6d). One possible explanation is that the perceived motion direction of the plaid has a stronger impact on MAE direction in the attended condition than in the non-attended condition. In particular, this perceived direction conforms to the so-called intersection of constraints^53^ which for our stimulus is a direction slightly different from that of the vector sum of the two gratings’ motions (see Fig. 6b). The MAE direction we observed in the unattended condition is close to opposite to the direction of the vector sum^13,20^ (see Fig. 6c), but in the attended condition the MAE direction was closer to opposite to the ‘intersection of constraints’ motion direction that is observed during adaptation, suggesting that perceived adapter direction impacts MAE direction more strongly when the adapter is attended (see Fig. 6d).

If we accept the notion that inattention promotes patchwork rivalry, then the profound impact of inattention on binocular rivalry might not, as has been proposed^43,54^ lie in attention’s putative contribution to suppressing one of the monocular signals. Instead, attention’s role in achieving unitary perception here would lie in its contribution to binding features across space, for instance based on^55^. One appealing aspect of this idea is related to the recognized^9^ but unexplained observation that other forms of perceptual bi-stability are not impacted as strongly by inattention. Because patchwork dominance, similarly, is much more common in binocular rivalry than in other forms of bi-stability, the present notion would provide a neat solution to that mystery as well.

## Supplementary information

Supplementary Information
