# Supplementary material for: Dichoptic vision in the absence of attention: neither fusion nor rivalry
Source: Sci Rep. 2019 Sep 9;9:12904. doi: 10.1038/s41598-019-49534-x (PMC6733948; doi:10.1038/s41598-019-49534-x)
Supplement: Supplementary file 1 — Supplementary Information [file 41598_2019_49534_MOESM1_ESM.pdf]

Supplementary Information for

Dichoptic vision in the absence of attention: neither fusion nor rivalry

Cheng Stella Qian[1], Sam Ling[2, 3], Jan W. Brascamp[1, 4]

[1]Department of Psychology, Michigan State University

[2]Department of Psychological and Brain Sciences, Boston University

[3]Center for Systems Neuroscience, Boston University

[4]Neuroscience Program, Michigan State University

		Conditions					
		Attended Dichoptic Pre.	Attended Superimposed Pre.	Attended Sequential Pre.	Unattended Dichoptic Pre.	Unattended Superimposed Pre.	Unattended Sequential Pre.
Experiment 1a							
Magnitude	Mean	0.19	0.21	0.13	0.14	0.17	0.09
	95% CI	0.09	0.14	0.05	0.06	0.07	0.03
		0.30	0.28	0.20	0.23	0.26	0.16
Initial Angle	Mean	103.48	113.62	82.39	89.99	111.09	76.88
	95% CI	95.56	103.57	53.89	79.23	99.05	53.24
		111.41	123.67	110.88	100.76	123.12	100.52
Horizontal component of MAE	Mean	-0.04	-0.11	0.00	-0.01	-0.05	0.00
	95% CI	-0.06	-0.15	-0.02	-0.02	-0.08	-0.01
		-0.01	-0.06	0.01	0.00	-0.03	0.00
Vertical component of MAE	Mean	0.19	0.17	0.12	0.14	0.16	0.09
	95% CI	0.09	0.10	0.04	0.05	0.07	0.03
		0.29	0.24	0.20	0.23	0.25	0.16
Experiment 1b							
MAE Angle	Mean	94.37	122.65	98.35	93.24	123.13	101.33
	95% CI	84.32	90.25	76.14	85.79	84.91	66.73
		104.42	155.05	120.56	100.69	161.36	135.94
Experiment 2							
SAE Angle	Mean	8.61		1.16	4.52		0.97
	95% CI	1.86		-0.66	2.15		-1.05
		15.36		2.97	6.89		2.99

Table 1. Descriptive statistics of all conditions for each experiment.

		Repeated Measures ANOVA with both Attention and Adaptation			Post hoc analysis		
		Attention Effect	Adaptation Effect	Interaction	Dichop vs Super	Dichop vs se	super vs seq
Experiment 1a							
Magnitude	F value	F (1,7) = 22.86**	F (2,14) = 15.97***	F (2,14) = 0.14		**	***
	effect size	0.76	0.7	0.02			
	MSE	0.001	0.002	0.004			
Initial Angle	F value	F (1,7) = 1.16	F (2,14) = 9.33**	F (2,14) = 0.29	**	*	*
	effect size	0.14	0.57	0.04			
	MSE	534.81	459.23	436.05			
Horizontal component of MAE	F value	F (1,7) = 6.57*	F (2,14) = 32.96***	F (2,14) = 3.25	*	**	*
	effect size	0.48	0.83	0.32			
	MSE	0.001	0.001	0.001			
Vertical component of MAE	F value	F (1,7) = 24.34**	F (2,14) = 22.24**	F (2,14) = 1.03		**	**
	effect size	0.78	0.88	0.26			
	MSE	0.000	0.001	0.002			
Experiment 1b							
MAE Angle	F value	F (1,3) = 0.15	F (2,6) = 6.44*	F (2,6) = 0.64			
	effect size	0.05	0.68	0.18			
	MSE	24.49	292.73	13.53			
Mixed Effect Models							
MAE Angle	F value	F (1,483) = 0.11	F (2,483) = 115.79***	F (2,483) = 0.11	***		***
Experiment 2							
SAE Angle	F value	F (1,7) = 1.75	F (1,7) = 10.15*	F (1,7) = 2.39	-	-	-
	effect size	0.2	0.59	0.25			
	MSE	20.96	23.85	12.79			

		Repeated Measures ANOVA with the Unattended Conditions	Post hoc analysis of the unattended conditions		
		Adaptation Effect	Dichop vs Super	Dichop vs seq	super vs seq
Experiment 1a					
Magnitude	F value	F (2,14) = 11.64**		**	**
	effect size	0.62			
	MSE	0.001			
Initial Angle	F value	F (2,14) = 6.41*	*		*
	effect size	0.49			
	MSE	371.71			
Horizontal component of MAE	F value	F (2,14) = 19.61***	**		**
	effect size	0.74			
	MSE	0.001			
Vertical component of MAE	F value	F (2,14) = 9.53**		**	*
	effect size	0.58			
	MSE	0.001			
Experiment 1b					
MAE Angle	F value	F (2,6) = 7.54*			
	effect size	0.72			
	MSE	124.42			
Mixed Effect Models					
MAE Angle	F value	F (2,486) = 116.48***	***		***
Experiment 2					
SAE Angle	F value	F (1,7) = 7.16*	-	-	-
	effect size	0.51			
	MSE	7.026			

Table 2. The inferential statistics of all the experiments. Repeated measure ANOVA with both attention and adaptation factors were conducted for each experiment. To further understand the unattended conditions, repeated measures ANOVA for the unattended conditions were also conducted for each experiment. Post hoc analysis all had LSD correction. Mixed effect modeling was conducted to Experiment 1b. The effect size is partial eta square. *** $p < 0.001$; ** $p < 0.01$; * $p < 0.05$; - doesn't apply
